# Use of intravenous immunoglobulin in the Department of Neurology at Ninewells Hospital, 2008–2009: Indications for utilization and cost-effectiveness

**DOI:** 10.4103/0972-2327.74199

**Published:** 2010

**Authors:** Jonathan O’ Riordan, Robert J. Swingler, Naveed M. Malek

**Affiliations:** Department of Neurology, Ninewells Hospital, NHS Tayside, Dundee, United Kingdom

**Keywords:** Blood transfusion service, Department of Health, United Kingdom, Intravenous immunoglobulin

## Abstract

This study was designed to identify the indications for prescription of intravenous immunoglobulin (IVIg) in neurology and the cost effectiveness of this therapy.

**Objectives:**

IVIg is a relatively costly therapy and the annual budget spent on providing this therapy for various indications at Ninewells Hospital was close to £1.5 million. In today’s economic times, a cost–benefit analysis of all therapies is prudent. This is of relevance to countries in the developing world as well where perhaps not everybody could afford such cost-intensive therapy.

**Materials and Methods:**

We audited 2 time periods over 12 months each in 2004–2005 and 2008–2009 to look at the patterns of utilization of IVIg over these periods. We searched the literature for alternative and cost-effective therapies for the most common indications for use of IVIg.

**Results:**

Fiscal costs on prescription of IVIg have rocketed up by almost 300% in this Neurology Department comparing data from 2004–2005 vs 2008–2009 and this is disproportionate to the increase in the annual admission rate (bed usage), partly because of the soaring costs of the drug available in the market and also because of the increased prescription of IVIg for numerous indications where clinical trials data are yet not so robust.

**Conclusion:**

We have looked at the cost of alternative therapies and offer some proposals that if implemented could potentially save £330,000 annually from the health budget at this NHS Trust. Perhaps similar models could evolve for better cost-effective utilization of IVIg in countries in the developing world where health budgeting is more acutely relevant.

## Current Literature

High-dose intravenous immunoglobulin (IVIg) has emerged as an important therapy for various neurologic diseases. It is certainly used for many more indications than what it is actually licensed for use in Neurology. Different interpretations of clinical trials data result in differing opinions among Neurologists about the long-term cost-effectiveness of this therapy in certain conditions in different parts of the world. Although it undoubtedly shows remarkable benefit in conditions, such as Guillian–Barré syndrome (GBS), the long term cost effectiveness of IVIg compared with that of alternative therapies in chronic inflammatory demyelinating polyradiculoneuropathy (CIDP); and issues about IVIg’s safety, cost, and mechanisms of action have raised concern among some practitioners.

## Lacunae

To explore alternative forms of cost-effective therapies, this article examines the prescription of IVIg in a typical UK Neurology Department and compares it with the National guidelines to see if some cost-saving measures could be adopted without compromising on the quality of treatment.

IVIg is a relatively costly therapy and the annual budget spent on delivering this treatment at Ninewells Hospital for various indications (not limited to Neurology) is close to £1.5 million (includes the drug cost, staffing costs, and hospital bed occupancy costs).

It costs an average of £7700 (total cost; includes cost of medication, bed costs and staffing costs) for a 5-day course of IVIg treatment and is utilized for many indications in the Neurology Ward in addition to its Licensed use in the UK in Neurology practice, that is, Guillian Barre Syndrome.

This audit was designed to see how effectively resources had been utilized in the Neurology Ward at Ninewells Hospital and to assess if the costs incurred by the Tayside NHS Trust on this account could be reduced without compromising on the quality of treatment.

This could equally apply to secondary/tertiary care centers in other developing countries, such as India, where many people probably cannot afford this sort of cost-intensive treatment, particularly when alternative therapies for some of the indications (see below) could be as effective yet cost a fraction of the treatment costs incurred on IVIg.

IVIg was produced by the blood transfusion service (BTS) locally/regionally with no major monetary costs to the Tayside NHS Trust as it was obtained from pooled human plasma available to the blood banks as a byproduct from fractionation of whole blood to produce Packed Red Blood Cells and Platelet concentrates for transfusion; however, since 1999, as concerns over contamination of domestic blood supplies in the context of new variant Creutzfeldt–Jacob disease (nvCJD) grew, the focus has shifted to buying the commercial preparation off the shelf from the international market in the country as a whole and since March 2006 this has been the trend at NHS Tayside as well.

The supply of human immunoglobulin in the international market has become reduced in recent years, drug costs have as a consequence risen to £3960 for a typical 5-day IVIg course for a person with an average body weight of 60 kg (excluding hospital staffing and bed costs) and the current trend in the international market seems to be heading towards further increase in costs.[1]

These costs are now borne by individual NHS Trusts and represent a significant cost to the NHS Trusts (£796,174 annually at Ninewells Hospital for purchase of the drug off the shelf and an equivalent amount is spent on costs of bed usage and staffing).The cost of prescribing this drug on the ward costs as much as buying the drug from the market (in this hospital).

It would be reasonable to say that health care budgeting should be relevant both to the NHS and to the individual Trusts. Cost-saving measures in today’s economic times therefore would make sense and this would be as relevant in developing countries, such as India, as it would be in countries, such as the United Kingdom.

The other issue that would be pertinent to mention here is the recognized fact that not all prescriptions of IVIg are based on robust evidence of effectiveness. This is equally true for the use of IVIg in Neurology as it is for other medical specialties.

## Materials and Methods

We audited 2 time periods over 12 months each in 2004–2005 and then in 2008–2009.

Data on use of IVIg were collected from the BTS computer records at Ninewells Hospital. Patient data were collected from the patients’ hospital Case Notes. Bed costs and staffing costs were obtained from the Finance Department at Ninewells Hospital.

### Setting

Ninewells Hospital in Dundee, Scotland, is a Tertiary Care Hospital with a Neurosciences Department where Neurologists, Neurosurgeons, Neurophysiologists, Liaison Psychiatrists and Neuropsychologists work in close collaboration to serve a catchment area of 420,000 people in the South East of Scotland.

Consultant staff in the department: Number of Consultant Neurologists, 5; number of Consultant Neurosurgeons, 3; number of Liaison Consultant Psychiatrists, 1; number of Consultant Neuropsychologists, 1 and number of Consultant Neurophysiologists, 1.

Total Number of Neurology Admissions: 761 patients in 1 year.

Total days of bed occupancy: 4579 occupied patient bed days during this period. Average period of stay for 1 patient: 6 days in total.

## Data Presentation

### Time period

From 04/01/04 to 03/31/05 and from 09/01/2008 to 08/31/09 [Tables 1 and 2].

**Table 1:** Bed usage (days) at Ninewells Hospital for IVIg as per clinical indication

Patient diagnosis	Audit 2004-2005*	Audit 2008-2009**
CIDP	114 patient days	126 patient days
MG	4 patient days	80 patient days
GBS	5 patient days	35 patient days
Dermatomyositis	24 patient days	0 patient days
Stiff Person syndrome	3 patient days	0 patient days
Other uses	42 patient days	84 patient days
Cumulative days of bed usage/year	192 patient days	325 patient days
Total cost of bed usage at that time	£88,704	£243,100

IVIg, intravenous immunoglobulin; CIDP, chronic inflammatory demyelinating polyradiculoneuropathy; MG, myasthenia gravis; GBS; Guillian–Barré syndrome. Other uses: Paraneoplastc axonal neuropathy, ganglionopathy, other sensorimotor neuropathy (axonal), neuromyotonia, progressive cerebellar ataxia, and diffuse polyradiculopathy of undetermined etiology.

* From 04/01/04 to 03/31/05 and

** from 09/01/08 to 08/31/09.

**Table 2:** Cost of prescription of IVIg in Neurology Department based on clinical diagnosis (2008–2009) assuming that IVIg was available free of cost to the Trust in 2004–2005 from blood transfusion service for reasons mentioned earlier

Patient diagnosis	Number of patients	Age group/age (years)	Total admissions	Admission emergency/elective	Costs of medication alone
CIDP	9 (5 M, 4 F)	38–82	28	Elective	£117,475.5
GBS	7 (4 M, 3 F)	32–93	7	Emergency	£36,234
MG	8 (4 M, 4 F)	26–86	16	Emergency (6) Elective (10)	£51,414
MMN	1 (F)	68	11	Elective	£19,602
Axonal neuropathy (paraneoplastic)	3 (2 M, 1 F)	61–75	4	Elective	£15,259
Axonal neuropathy (inflammatory/progressive)	3 (2 M, 1 F)	60–79	4	Elective	£15259
Ganglionopathy	1 (M)	51	2	Elective	£7629
Progressive cerebellar ataxia	1 (F)	61	1	Elective	£3815
Polyradiculopathy	1 (F)	69	2	Elective	£7629
Neuromyotonia	1 (M)	89	1	Elective	£3815
Probable CIDP	1 (F)	48	1	Elective	£3815
Total	37		78	Elective (65) Emergency (13)	£281,947.5

IVIg, intravenous immunoglobulin; BTS, blood transfusion service; CIDP, chronic inflammatory demyelinating polyradiculoneuropathy; MG, myasthenia gravis; MMN, multifocal motor neuropathy; GBS; Guillian–Barré syndrome.

## Discussion

IVIg is used in Neurology for many indications, which have been classified by the Department of Health (DOH), UK, as follows.

### Licensed indication

Guillian–Barré syndrome.

### Other indications

- Chronic Inflammatory Demyelinating Neuropathy (CIDP)
- Dermatomyositis
- Lambert–Eaton myasthenic syndrome
- Multifocal motor neuropathy (MMN)
- Myasthenia gravis (MG) crisis
- Paraprotein-associated demyelinating neuropathy
- Rasmussen encephalitis
- Stiff person syndrome

These are diseases for which there is a reasonable evidence base but where other treatment options are available.

### Grey indications

- Acute disseminated encephalomyelitis
- Acute idiopathic dysautonomia
- Autoimmune diabetic proximal neuropathy
- Bickerstaff’s brain stem encephalitis
- Cerebral infarction with antiphospholipid antibodies
- Central nervous system vasculitis
- Intractable childhood epilepsy
- Neuromyotonia
- PANDAS (pediatric autoimmune neuropsychiatric disorders associated with streptococcal infection)
- Paraneoplastic disorders
- POEMS (polyneuropathy, organomegaly, endocrinopathy, monoclonal gammopathy, skin changes)
- Polymyositis
- Potassium channel antibody-associated nonlimbic encephalitis
- Vasculitic neuropathy

Grey indications are those for which the evidence base is weak.

### Black indications

- Adrenoleukodystrophy
- Alzheimer’s disease
- Amyotrophic lateral sclerosis
- Chronic fatigue syndrome
- Critical illness neuropathy
- Inclusion body myositis
- Multiple sclerosis

The prescription of IVIg for these indications is not appropriate.

IVIg can be an expensive therapeutic choice in disease states where other therapeutic options may be available. Even if there are data that support the potential efficacy of IVIg, its use should still be carefully considered, not only because of supply issues, but because of potential and often individual risks,for example, anaphylactoid reactions to IVIg given to pregnant women can lead to acute fetal compromise. In the 1980s and 1990s, cases of hepatitis C transmission were reported with IVIg. Since the standardization of viral inactivation steps, there might have been no hepatitis B or C transmissions, but there is no place for complacency because of the possibility of unknown as well as novel viruses and other infectious agents, particularly nvCJD; vigilance is required and it would be wise to be cautious, and indiscriminate prescription should be checked and strongly discouraged.

Now looking at the results of the audits done in 2004–2005 and 2008–2009, it is evident from Table 1 that annual admission rate (bed usage) in the Neurology Department has gone up by 60% year on year and the cost of bed usage has also increased since then from £462/day to £ 748/day, which represents an increase of 60% in itself, but the overall cost to the Trust in terms of bed occupancy costs alone has increased by almost 300% from £88,704 to £243,100 [Table 1], which is very significant.

IVIg, as pointed out earlier, cost essentially nothing extra to the Tayside NHS Trust when it was produced locally but now when Tayside NHS Trust has to buy the commercial product off the shelf form the International markets (since March 2006), this represents an additional cost of £281,947.5 [Table 2] annually to the Neurology Department alone.

The other thing of note in the data above was that patients with polymyositis were not admitted for IVIg treatment in the time period 2008–2009 compared with 2004–2005, which is in line with the DOH guidelines of 2008; however, patients admitted electively under the group label “others” in Table 1 included patients with paraneoplastic neuropathy (axonal), other sensorimotor neuropathies (axonal), progressive cerebellar ataxia, ganglionopathy, neuromyotonia, and so on,; these patients availed 84 days of total bed usage at Ninewells Hospital at a cost of £57,222 for prescribing IVIg and a total cost £113,322 (including £56,100 for bed usage) [Tables 1 and 2].

The number of emergency admissions (Guillian–Barré syndrome + MG) increased significantly from 2(2004–2005) to 15(2008–2009) patients, whereas the number of elective admissions (CIDP + MMN) increased slightly from 114 days of cumulative bed usage annually (2004–2005) to 126 days of cumulative bed usage (2008–2009) [Table 1].

Only 1 patient in the cohort we looked at died. He had MG but at the time of his death neither was he receiving IVIg nor was he in this hospital. The autopsy report revealed that the cause of death was pulmonary embolism.

Most patients reported a subjective improvement after therapy. In the case of Myaesthenic and GBS patients improvement in strength was objective. In patients with CIDP, subjective and objective improvement was noted but there could possibly be a placebo effect as well in terms of subjective well being particularly in patients with predominantly sensory symptoms, which is difficult to delineate from pharmacologic effects of the drug.

People who were at baseline on pyridostigmine for MG remained on this drug and people who were at baseline on immunosuppressants for MG or CIDP, including mycophenolate, azathioprine, or prednisolone, remained on those drugs. No medications were stopped or altered because of the IVIg therapy.

Analyzing the data from the audit, our conclusions and where relevant *recommendations* to conserve costs to the Tayside NHS Trust and to the NHS overall, although this could apply to other countries as well with appropriate modifications in their regional contexts, from the point of view of use of IVIg in Neurology would be:

1. IVIg is used in Neurology for indications other than its Licensed indication for which there is randomized controlled trial (RCT) evidence of benefit in the short term without changing long-term outcomes, such as CIDP, but it is also used for indications, such as Paraneoplastic Axonal Neuropathy (noted above), for which there is yet no consensus if it is of benefit either in the short term or long term; these are conditions where Physician preference for indication for use of IVIg are more variable. A consensus-based approach among Neurologists on standard indications for use reflecting the DOH guidelines would be highly desirable. This could potentially save £113,322 (medication + bed usage costs) that were spent last year on these indications in the Neurology Department at Ninewells Hospital, if consensus could be achieved amongst Neurologists on restricting IVIg use to RCT evidence-based indications.[1]
2. There are alternative therapies for conditions, such as CIDP, which could possibly have a similar efficacy to IVIg based on RCT evidence, these could include intravenous steroid infusions for CIDP from the point of view of a cost-effective approach, if the efficacies of these therapies are similar in the short- and long terms, it could lead to significantly trimming the costs of therapy for this particular indication provided drug side effects do not outbalance the intended benefits or can be prevented.[2]
3. If patients with chronic conditions who come for regular elective admissions throughout the year could receive their IVIg infusions in the community, that is, GP surgeries, that could potentially save 148 days of hospital bed usage (CIDP and MMN combined) costing £110,704 (average cost of bed usage is £748/day at current estimates). We could see from the new data we have collected that this recommendation has/could not be implemented because of either technical or safety considerations at present; however, this should be open for review and discussed with all the GPs.[3]

For an average person (60 kg), an IVIg course over 5 days costs £3960 (excluding bed usage and staffing costs), 5 sessions of plasma exchange costs £5780(excluding bed usage and staffing costs), and a 3-day 1 g IV methyl prednisolone course for cost comparison costs £52 (excluding bed usage and staffing costs). There is a potential to save £110,000 annually if this recommendation could be accepted.

Taken together, there is a potential to save £330,000 annually in the Neurology Department alone from a total annual budget of about £1.5 million that is spent on IVIg (cost of medication + bed costs) at Ninewells Hospital (all departments taken together) at current estimates and possibly more if other departments, such as Hematology, Oncology, Rheumatology, and Dermatology where IVIg is prescribed for other indications could come up with their own recommendations.[4]

## Conclusion

If all the 3 recommendations made above could be implemented, in particular recommendation 2 and 3 as these are more policy-based decisions than logistic decisions, costs to the NHS Trust or to their equivalents in other countries,could well be reduced significantly and this money could possibly be better utilized for other unmet health needs; we assume this would be relevant to the developing countries as well.

We could all do our bit in contributing to health care rationing and saving money, perhaps more consensus-based decisions on the Neurology Ward about the best utilization of limited resources could lead to objective policies in which long-term goals could be as important as short-term gains.

The DOH, UK, acknowledges that there is a clear need to provide guidance on the appropriate use of IVIg and a framework for the promotion of evidence-based clinical practice to help improve consistency in patient care. There could be comparable standardized guidelines in other countries, such as India, which would be tailored to the specific health care needs of its people in its own social, economic, and health contexts and this could be as relevant in any country as it is in the United Kingdom. The overall goal of any guideline or document issued in this context would be to ensure best practice in the use of IVIg across all indications, based on the available RCT evidence and consensus expert opinion.
